# Equitable data sharing in epidemics and pandemics

**DOI:** 10.1186/s12910-021-00701-8

**Published:** 2021-10-06

**Authors:** Bridget Pratt, Susan Bull

**Affiliations:** 1 Queensland Bioethics Centre, Australian Catholic University, 1100 Nudgee Rd., Brisbane, Australia; 2 Centre for Health Equity, School of Population and Global Health, University of Melbourne, Melbourne, Australia; 3 The Ethox Centre, Nuffield Department of Population Health, University of Oxford, Oxford, UK

**Keywords:** Ethics, Data sharing, Covid-19, Pandemic, Epidemic, Utility, Equity

## Abstract

**Background:**

Rapid data sharing can maximize the *utility* of data. In epidemics and pandemics like Zika, Ebola, and COVID-19, the case for such practices seems especially urgent and warranted. Yet rapidly sharing data widely has previously generated significant concerns related to *equity*. The continued lack of understanding and guidance on equitable data sharing raises the following questions: Should data sharing in epidemics and pandemics primarily advance utility, or should it advance equity as well? If so, what norms comprise equitable data sharing in epidemics and pandemics? Do these norms address the equity-related concerns raised by researchers, data providers, and other stakeholders? What tensions must be balanced between equity and other values?

**Methods:**

To explore these questions, we undertook a systematic scoping review of the literature on data sharing in epidemics and pandemics and thematically analyzed identified literature for its discussion of ethical values, norms, concerns, and tensions, with a particular (but not exclusive) emphasis on equity. We wanted to both understand how equity in data sharing is being conceptualized and draw out other important values and norms for data sharing in epidemics and pandemics.

**Results:**

We found that values of utility, equity, solidarity, and reciprocity were described, and we report their associated norms, including researcher recognition; rapid, real-time sharing; capacity development; and fair benefits to data generators, data providers, and source countries. The value of utility and its associated norms were discussed substantially more than others. Tensions between utility norms (e.g., rapid, real-time sharing) and equity norms (e.g., researcher recognition, equitable access) were raised.

**Conclusions:**

This study found support for equity being advanced by data sharing in epidemics and pandemics. However, norms for equitable data sharing in epidemics and pandemics require further development, particularly in relation to power sharing and participatory approaches prioritizing inclusion. Addressing structural inequities in the wider global health landscape is also needed to achieve equitable data sharing in epidemics and pandemics.

**Supplementary Information:**

The online version contains supplementary material available at 10.1186/s12910-021-00701-8.

## Background

Open science and data sharing are increasingly becoming accepted norms of good research, and data sharing has played a critical role in the development of public health responses to the COVID-19 pandemic. A key example is the early sequencing and sharing of the SARS CoV-2 viral genome by Chinese scientists on 8 January 2020 [1] and subsequent sharing of the genomes of variants of concern. Drawing on such data, the subsequent scale and pace of international COVID-19 vaccine research and rollout has been unprecedented.

The importance of sharing data to address global health priorities, including informing responses to outbreaks and epidemics, is widely recognized [2]. In the context of COVID-19, widespread mandates for data sharing have been developed by key stakeholders, including national governments, global health NGOs, journals, research funders and research institutions. These mandates have been accompanied by significant developments in the policies and infrastructures required to support such sharing [3].

International data sharing mandates stress the importance of maximizing the *utility* of data, whilst recognizing the importance of ensuring that approaches to data sharing are *equitable* [2]. The rapid sharing of data as widely as possible is often recognized as key to maximizing data utility. In epidemics and pandemics, the case for such practices seems especially urgent and warranted to develop much-needed vaccines, therapeutics, and diagnostics. However, data sharing norms prioritizing utility have previously generated concerns related to equity. Current data sharing mandates, for example, have been considered insufficiently responsive to low and middle-income country (LMIC) perspectives, interests, and contexts [4–6]. The disproportionate availability of resources for data analysis in wealthy institutions, for example, may result in researchers from high-income countries (HICs) being well equipped to make use of data shared from LMICs, while the reverse is less often true [7].

In 2011, 17 health research funders from across the world jointly affirmed the need to share data in ways that are equitable [8]. To date, however, there has been limited discussion about what equity and social justice require in the context of data sharing [9]. Consequently, questions still arise about what equitable sharing comprises, and how to balance tensions between equity and other important values like utility [10, 11]. Consideration of how data should be shared equitably is arguably particularly important in contexts of epidemics and pandemics, given the critical need to rapidly and effectively develop responses to their associated substantive and inequitable impacts on populations’ health and wellbeing. Key questions include: What values are thought to be advanced by sharing data in epidemics and pandemics? What norms should underlie data sharing in epidemics and pandemics? Do these norms promote equity and address the equity-related concerns raised by researchers, data providers, funders, and other stakeholders? How should tensions between equity and other values, such as utility, be addressed?

To investigate these questions, we undertook a systematic scoping review of the literature on data sharing in epidemics and pandemics and thematically analyzed identified literature for its discussion of ethical values, norms, concerns, and tensions, with a particular (but not exclusive) emphasis on equity. We wanted to both understand how equity in data sharing was being conceptualized and draw out other important values and norms for data sharing in epidemics and pandemics. In the paper, we primarily focus on sharing health-related datasets for public health and research purposes. However, discussions of sharing potential outputs of data sharing, including research findings and health interventions,^1^ are also addressed to the extent that they are considered in conjunction with data sharing in the literature.^2^ Likewise, we have included discourses about sample sharing where these are addressed in tandem with data sharing, but a substantive consideration of sample sharing is beyond the scope of this paper. We report on the types of health-related data considered important to share in epidemics and pandemics, values thought to be promoted by sharing such data, norms for sharing data that promote those values, equity-related concerns, and tensions between identified values. We conclude by reflecting on whether identified equity-related norms are sufficient to address identified equity-related concerns and to promote equitable data sharing in epidemics and pandemics.

## Methods

### Literature review

Scoping reviews seek to identify literature relevant to the research objective and may include a variety of article formats [12, 13]. This scoping review sought to identify key concepts and characterizations of the values promoted by data sharing in the literature on health-related data sharing in outbreaks, epidemics, and pandemics. Two searches were undertaken in June and July 2020, comprising a formal literature review and a review of literature in the Epidemics Ethics Database. The latter yielded not only editorials, articles, and commentaries in peer-reviewed journals but also news articles, blogs, reports, and guidance and policy documents.

**Fig. 1:**
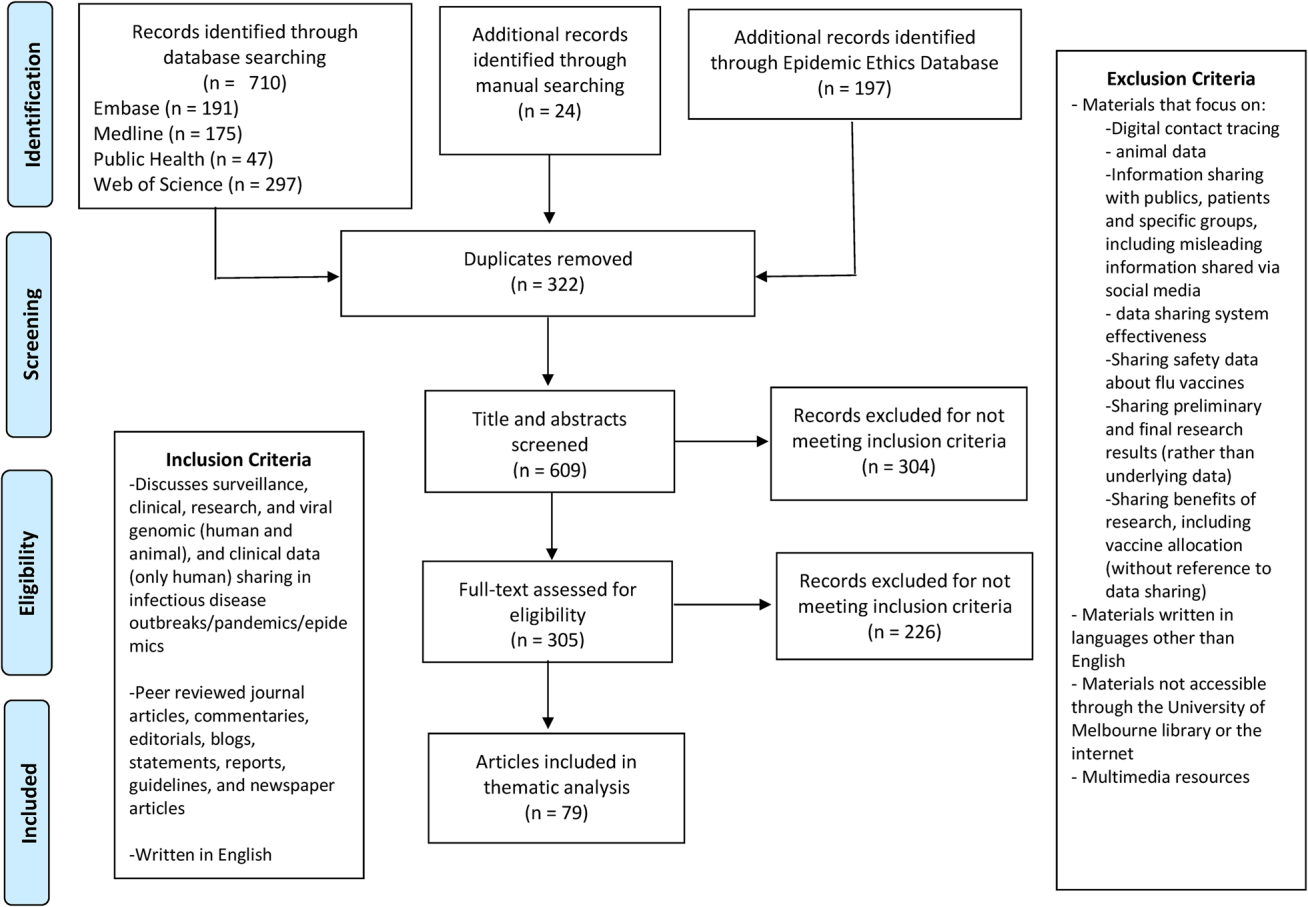
PRISMA diagram

In conducting the formal literature search, two categories of search terms were used: (1) data sharing and (2) infectious disease outbreaks/epidemics/pandemics, informed by a prior systematic scoping review of data sharing ethics [10] and informatics expertise at the University of Melbourne Brownless Biomedical Library. The two categories of search terms were further developed for this study through an iterative process, where combinations of controlled vocabulary and key words were piloted in Ovid Medline. The following four databases were searched for relevant studies: Embase (OvidSP) [1974-present], Global Health (OvidSP) [1973-present], MEDLINE(R) (OvidSP) [1946-present], and Science Citation Index (Web of Science Core Collections, Thomson Reuters) [1945-present]. The searches were conducted on 7 June 2020. No publication date limits were applied. (The full search strategy is available in Additional File 1). In total, 388 citations were identified in the formal literature after de-duplication between the four searches (Fig. 1). References were imported into bibliographic software (Endnote X9).

The Epidemic Ethics Database (https://epidemicethics.tghn.org/resources) compiles resources in relation to the ethical issues arising out of global health emergencies, with a current focus on the COVID-19 pandemic. Three separate, key word searches of the Epidemic Ethics Database were performed: (1) data and sample sharing, (2) surveillance & apps & AI, and (3) global health justice. The selection of these key words reflected key word options within the database itself and the overall study aim of exploring what comprises equity in data sharing during infectious disease epidemics and pandemics. The searches were conducted on 12 July 2020. No publication date limits were applied. In total, 197 articles were identified (Fig. 1).

### Literature screening

A matrix of inclusion and exclusion criteria was developed to inform screening (see Fig. 1).

All titles and abstracts from the formal literature search were assessed by the first author. 84 papers were identified for full text screening, of which 42 were subsequently excluded (Fig. 1). The full text of all 197 resources from the Epidemic Ethics Database were screened because many identified documents, such as editorials and policy statements, that came without abstracts. Of the 197 articles, 184 were subsequently excluded (Fig. 1). An additional 24 articles were added based on literature the authors were familiar with and handsearching. To ensure rigor, a sample of 15% of full text articles included in the study was co-reviewed by the last author. All full text articles that were considered ‘possible inclusions’ by the first author were also reviewed by the last author and a mutual final decision was then made on their inclusion. In total, 79 articles (42 from the formal search, 13 from the Epidemic Ethics Database, and 24 from handsearching) were identified by the scoping review. A full list is provided in Additional File 2.

### Thematic analysis

The 79 full-text articles that met the inclusion criteria were thematically analyzed using the approach described by Braun and Clarke [14]. That approach involves the following main steps: (1) familiarization with the data, (2) generating a coding framework, (3) searching for categories and sub-categories and (4) reviewing categories and sub-categories. A broad coding framework was developed relying on a priori categories that were selected based on the study’s research question. The five main categories were: (1) types of data being shared, (2) values furthered by data sharing, (3) norms of data sharing (how should data be shared), (4) equity-related concerns raised about data sharing, and (5) tensions between values/norms that arose when sharing data. The authors independently coded articles and developed sub-categorizations within those categories that were drawn inductively from the data. Similarities and differences between the two sets of sub-categorizations were discussed and addressed during collaborative development of the coding framework by both authors. Coding of full-text articles was then performed by the first author using NVivo Version 12.

## Results

In this section, we report findings on the types of data considered important to share during epidemics and pandemics, the values thought to be promoted by data sharing in such contexts, norms for sharing data, equity-related concerns, and tensions between identified values. The norms and concerns discussed in this paper relate to sharing traditional surveillance, clinical, viral genomic, and research data. Discussions about norms and concerns relating to curating and sharing digital surveillance data in the context of COVID-19 were so distinct that they are discussed in a separate paper.

### Types of data to share during epidemics and pandemics

Four types of health-related data were discussed as important to share during infectious disease epidemics and pandemics: *surveillance, clinical, viral genomic, and research (biomedical and non-biomedical)*. Surveillance data included epidemiological data such as the number of confirmed cases, hospitalizations, and reported deaths as well as data on confirmed cases: travel history, location of infection, reported onset dates, and basic demographics. Clinical data comprised individual case data, including physiologic, laboratory, imaging, treatment, and outcome data. Viral genomic data included viral genomic sequencing and protein structure data, at times with associated phenotypic/clinical outcome data. The importance of sharing this data both with health authorities for public health purposes and with researchers for research purposes was recognized.

Biomedical research data primarily referred to data collected and materials (such as analysis strategies) developed during research into the characterization, diagnosis, and treatment of infectious diseases and during vaccine development, including clinical trial data and drug repurposing data. Non-biomedical research data referred to research data on the social and economic impacts of epidemics and pandemics on health and wellbeing, including for populations considered to be disadvantaged, vulnerable, or marginalized during such emergencies. Their sharing was discussed far more infrequently than that of biomedical research data.

### Value of sharing data during epidemics and pandemics

Sharing health-related data for public health and research purposes during epidemics and pandemics was described as expressing or advancing several values in the identified literature (Table 1). *Utility* was by far the most commonly discussed, in addition to *equity and health justice, reciprocity*, and *solidarity*.

**Table 1:** Values expressed or advanced by data sharing in epidemics and pandemics

Value	Description	Type of data	References
Equity and health justice	Sharing data is necessary to determine how infectious diseases affect different populations and social groups and how the epidemic or pandemic is exacerbating inequities	Research	[15, 16]
Reciprocity	Each country should do what it can to contribute through timely sharing of viruses and specimens, with the understanding that it can expect the same from the rest of the international community	Samples and viral genomic sequence data	[17, 18]
Solidarity	Data sharing and international collaboration across all scientific disciplines and between the public and private sectors reflects and addresses our common interests and shared vulnerabilities: *“Individual researchers should see their work not simply as parallel to or concurrent with the work of others, but rather as an integral part in a greater project of preventing disease and securing human flourishing”* (Langat et al. [19], p. 9). It is part of a global project of protecting human health and promoting human well-being	Research	[19, 20]
Utility	Sharing data maximises the public good and maximises benefits by saving lives and reducing suffering	All	[17, 21, 21–40]

The utility of data sharing in epidemics and pandemics was linked to multiple dimensions of maximizing benefits including:

- reducing suffering for current and future populations/generations,
- helping improve people’s quality of life during and after epidemics, and
- helping reduce the socio-economic impact of epidemics on societies.

Data sharing was primarily perceived as having the potential to reduce suffering now and during future epidemics and pandemics by averting illness and saving lives. A large emphasis was on sharing *biomedical* research data to develop interventions to diagnose, treat, and prevent disease. Sharing data from current epidemics and pandemics during and after their occurrence was also considered important to effectively respond to and potentially prevent future epidemics and thereby reduce the suffering of *future* generations. Reducing suffering was discussed in health-related terms, whereas the latter two dimensions of utility potentially speak to well-being more broadly. In the identified literature, however, neither well-being nor its various non-health-related components (e.g., respect, relationships and attachments, personal security) were explicitly mentioned.

### Norms of data sharing during epidemics and pandemics

Several data sharing norms were identified, not all of which mapped clearly on to the values as defined in the reviewed literature (Table 1). The identified norms include rapid, real-time sharing; capturing diversity; equitable access to data; capacity development; researcher recognition; fair benefits to data generators, data providers, study participants and communities, and source countries; and equitable global access to the benefits of research (Table 2). *Rapid, real-time data sharing* was by far the most-discussed norm in the context of epidemics and pandemics. Yet the importance of equity norms in data sharing in epidemics and pandemics was noted. For example:

*“in interviews, it became apparent that unless issues of equity are effectively addressed, data will not be shared at all.”* [41, p. 29]

**Table 2:** Norms for sharing data in epidemics and pandemics

Norm	Definition	References
Capacity development	Necessary skills and infrastructure should be developed. Requires immediate transfer of skills or to provide support systems to ensure that any locally produced health-related data can be rapidly generated and shared. Should guarantee equal infrastructure and resources to analyse the data. *“Only this parity in means and capacities of data analysis could confer justice in the use of the collected data.”* (da Costa and Leite [42])	[41, 41–45]
	Priority to LMIC investigators, especially those closest to an epidemic	
Capturing diversity	Data collected and shared should not be limited to epidemiological factors but also capture socio-economic differences that are known to drive disparities in infection rates	[15]
Data stewardship	Calls for gradual shift away from the culture of data ownership towards one of data stewardship for health-related datasets from human populations. Although countries are recognized to be the “key arbiters” of sharing data collected from their populations, *“in times of emergency, the onus should be placed upon the stewards of population- and individual-level data to justify if and why they are unwilling to share data for the good of public health.”* (Modjarrad et al. [25])	[25]
Equitable access to data	Data must be made available to all interested parties without cost or just at a level of recovering costs without profit	[2, 46, 47]
	Equitable access may mean giving priority access to research data to LMIC researchers or researchers from the source country/region	
Fair benefits to data generators (researchers), data providers, study participants and communities, and source countries	Individuals and communities that participate in research should have access to research outputs (such as vaccines) which result from the use of their data. Those to whom the data and samples relate also should not bear an undue burden for the benefit of others. These ethical imperatives are more pronounced when samples and data collected from potentially vulnerable populations are shared	[2, 21, 40]
	Benefit-sharing arrangements should ensure source countries can access any resulting vaccines or treatments	
	Researchers (in LMICs) should have the same opportunities (as those in HICs) to derive benefits from the data and samples that they have acquired themselves	
Equitable *global* access to the benefits of research	The benefits of science are a global common good. Globally, there should be equitable access to research outputs such as vaccines. Outputs should not be hoarded by any one country or organisation. Concept of a *“people’s vaccine”* has been raised	[18, 21]
	Particular consideration should be given to the specific needs of LMICs	
International collaboration	Global collaboration and cooperation; data sharing across national borders	[48, 49]
	Secondary users of data should make best efforts to collaborate with representatives of the originating laboratory or research team responsible for obtaining the specimen(s) and involve them in analyses and further research using such data	
National sovereignty	Recognizes the sovereign right of States over pathogen data and biological resources and their authority to determine the terms and conditions for accessing such resources	[50, 43]
Pre-publication and rapid review	Journals should support pre-publication data sharing and dissemination, and undertake rapid peer review	[3, 27, 34, 51]
Open access	Journals should make articles with datasets and findings that might have value in combating the epidemic/pandemic available to all, free of charge, as soon as is feasibly possible	[3, 41, 27, 52, 52–55]
Rapid, real-time data sharing	Researchers should release research protocols, materials, datasets and results related to epidemic/pandemics and make them publicly available without waiting for publication in scientific journals	[41, 18, 23, 42, 25, 29–31, 40–42, 58, 54–66]
	“*Researchers should be responsible for ensuring that shared results—even when preliminary—have undergone some quality control and are, therefore, sufficiently accurate*.” (Modjarrad et al. [25])	
	Information critical for public health should be shared with the World Health Organization, public health officials, the study participants and affected population, and groups involved in wider international response efforts before publication. Journals should state that they will only publish data-driven research arising from a public health emergency if it is accompanied by an explicit statement from authors that they have shared data and results with authorities and legitimate bodies responding to the emergency at the earliest possible opportunity	
Recognition	Secondary users of data must acknowledge the contribution of the data generator and/or the origin of the data. Primary researchers and data contributors should be credited in publications: *“Interviewees highlighted that agreed terms of use, collaboration and accreditation are especially needed for data generated during an active outbreak because those who are collecting the data and responding to the outbreak may have less time to be analysing data. If data is shared rapidly, the analysis may be completed by others before those who are treating patients have a chance to sit down at a computer, thus fostering inequity in opportunity to use shared data.”* (Pisani et al. [41], p. 37)	[2, 41, 21, 24, 19, 45, 67, 49, 38]
	Secondary users of data must also check whether data generators should be listed as *authors*—for example, some journals require authorship for anyone who designed, collected or analysed the paper’s intellectual content. LMIC researchers should get the authorship credit they deserve	
	Academic institutions should implement policies that recognize data sharing as an aspect used to help determine professional advancements	
	Journals should explore innovative ways of crediting significant intellectual input into research short of direct involvement in writing, and should consider publication policies that promote the inclusion of primary researchers in later re-analysis of their data	

In relation to surveillance data, capturing diversity means publicly reporting data on how epidemic and pandemic diseases affect different populations and social groups. Krieger [15] contends that:

*“testing and mortality data should be stratified by age, race, ethnicity, socioeconomic position, and gender. We also need data on type of work or unemployment, insurance status, sickness benefits, housing and homelessness, incarceration, nativity and citizenship status, sexual orientation, gender identity, and exposure to domestic violence*.”

In relation to virus sequence data, fair benefit sharing was discussed as being *in return* for data access. Researchers and public health authorities should be granted access to samples and data, provided the benefits arising from their use, including access to and distribution of affordable vaccines, diagnostics, and treatments to those in need, are shared in a timely manner [17, 18, 43, 50].

In relation to research data, ensuring fair benefits to study participants and communities was deemed especially critical when dealing with samples and data collected from potentially vulnerable populations. Sharing benefits with LMICs, especially those affected by the epidemic or pandemic, and giving their populations priority access to vaccines and treatments was also suggested as necessary [21]. Giving priority access to research data to LMIC researchers or researchers from the source country/region was also discussed:

*“An important principle of SLED [Sierra Leone Ebola Database] is to maintain access by African researchers, particularly from Sierra Leone.”* [46, p. 3]

*“We want to make sure that scientists working in countries where they don’t have a lot of resources have access to data and knowledge from high-income countries.”* [47]

Capacity development in LMIC research institutions, especially those closest to an epidemic, was highlighted as a priority to “ensure that every African country can produce local data to inform a local response” [44]. Recognition encompassed ensuring LMIC researchers, in particular, get the authorship credit they deserve when data are shared.

### Concerns about data sharing related to equity

Five concerns related to equity were discussed in the literature on data sharing during epidemics and pandemics. These concerns encompass data sharing not only as a transaction on its own but also as a key element of research pathways and partnerships. In addition to concerns about equitable data sharing, concerns about failing to generate and share non-biomedical health-related data relevant to epidemic and pandemic responses are reported.

#### Unfair benefits for LMIC researchers

Concern was voiced that asymmetries in capacity, infrastructure, and technology between HIC and LMIC researchers lead to an unfair distribution of benefits once data are shared [22–25, 42]. LMIC researchers who generate data or who are secondary users of shared data may be placed at a disadvantage due to their precarious access to equipment and other materials needed for analysis. They may also lack the time, necessary cutting-edge technology, and infrastructure for rapid data analysis, allowing HIC researchers to conduct studies and publish more quickly using the same dataset and to scoop their LMIC counterparts [42]. In epidemic contexts,

*“when data producers are on the ground working to stem an outbreak and are thus occupied with tasks more important than writing manuscripts, other scientists may analyze the released data, submit the resulting manuscript, and ultimately ‘scoop’ the data-producing lab.”* [22, p. 1235]

In such circumstances, the benefits of publishing first may then accrue primarily to secondary users of the data and/or HIC researchers. From researchers’ perspectives, publishing in scientific journals brings about two desired benefits: academic status and increased potential to acquire research funding [42]. In an epidemic or pandemic, the career benefits of publishing may even be magnified because the outbreak of a novel pathogen provides a unique opportunity to conduct truly groundbreaking research [68].

#### Lack of recognition for primary researchers

A second concern raised was a lack of credit, acknowledgement, and/or authorship accruing to data generators (primary researchers) by secondary users of shared data, especially for LMIC researchers [19, 21, 24–27, 56, 68–70]. This fear was discussed across several epidemics:

*“Some have been reluctant to do so [share H5N1 data] because they worry about intellectual-property rights or not receiving a fair share of the scientific credit.”* [26, p. 1224]

*“Researchers in Brazil who deposited Zika virus genome sequences in a public database felt they were not credited appropriately when another group used those sequences for a paper published 2 weeks later.*” [56]

#### Lack of benefit sharing with source countries and their populations

Concern was strongly voiced that secondary uses of shared samples and data could inform the development of commercialized medical products (vaccines, drugs, diagnostics) vital to combat a given epidemic/pandemic, which would not be accessible to source countries and their populations, including affected vulnerable social groups [17, 21, 25, 27, 69, 71–73]. During the H5N1 pandemic, for example, the Indonesian government learned that the virus samples it had shared were being used by pharmaceutical companies to develop new vaccines to be sold at commercial rates—rates that the Indonesian government believed it could not afford [17], demonstrating. In that context,

*“a new dimension of inequities in shared benefits from outbreak investigations is being recognized. The polarization is now magnified toward poor countries urged to donate ‘natural resources (clinical specimens, viruses and other microbes)’ versus technologically advanced industrial countries with private interests*.” [71]

Where governments retain their ownership of samples during epidemics and pandemics, as Indonesia did, it is not necessarily morally wrong [72]. A qualitative study also found that some stakeholders in the MERS-CoV epidemic in 2012 felt that researchers should respect countries’ sovereign rights:

*“Instead of seeing this as an impediment, they felt it should be seen as an opportunity to engage international partners in collaborative response efforts on an equal footing.”* [68, p. 11]

#### Stigmatization

Concern was expressed that data sharing during epidemics and pandemics could generate harms with equity implications: namely, stigma. Secondary use of research data, for example, can be stigmatizing at the group or population level in ways that only those with deep local knowledge may be able to anticipate or recognize [21]. In relation to surveillance data, Gasser et al. [67] similarly affirm they should be supplemented by robust safeguards, including analytical capacities to contextualize the data in order to avoid further stigmatization of underserved populations.

#### Failure to generate and share non-biomedical research data

While much of the identified literature focused on generating and sharing biomedical research data during epidemics and pandemics, there is concern that *non-biomedical research data* may not be sufficiently generated (i.e., funded) and shared in comparison. Bayram, Springer, Garvey, and Ozdemir [45] draw attention to the disparity between the enormous progress and investments made in digital technologies during COVID-19 and our understanding of those technologies’ societal dimensions. Abramowitz et al. [67] further note that WHO policy statements on data sharing privilege certain kinds of data, including surveillance data, genetic data, and clinical trial data. These priorities pre-empt other kinds of data collection and slow their integration and sharing during epidemics and pandemics. In the 2014–2016 Ebola outbreak, for instance, research on biological specimens took precedence, and funding, logistical support, and institutional support for data collection and data sharing did not keep pace with the expansion of social science demands [67].

Non-biomedical research generates vital information about the impact of epidemics and pandemics and their associated public health interventions (e.g., contact tracing, restrictions on people’s behaviors and movements) on those considered to be disadvantaged and marginalized and about whether policy and other interventions to address any negative effects are working. For example, it can generate data about the immediate and enduring effects of stay-at-home mandates on physical and sexual abuse and violence within households and whether and how these consequences can be mitigated [15]. It is thus essential that the research response in epidemics and pandemics include social science research and data sharing in order to ensure that health and social disparities are not worsened.

### Tensions

Tensions between identified values in the literature primarily arose in relation to promoting the values of utility and equity in sharing health-related data. First, promoting utility and generating public health benefits could be at odds with approaches that promote researchers’ interests:

*“Where there is a case for data and/or samples to be shared urgently for public benefit during an emergency, the imperative to help reduce suffering may at times make such protections for local researchers temporarily unjustifiable. If, in any particular case, immediate public benefit is sufficient to justify moving away from these supportive approaches prioritising the interests of local researchers, at least during the acute phase of an emergency, then fairness requires that researchers who are potentially disadvantaged have the opportunity to be fairly recognized by other means.”* [21, p. 211]

Second, promoting utility and generating public health benefits may also be in tension with national sovereignty, where it calls for restricting access to data in the name of national interest and/or national/global security. The exercise of ownership rights in the name of national interest or security can delay access to vital data during epidemics and pandemics. During the H5N1 pandemic, for example, Dutch and US authorities proposed withholding global scientific access to H5N1 viruses (in the form of sequence data) that scientific experts claimed were crucial for pandemic preparedness, because of concerns that these would provide bioterrorists a recipe for constructing a deadly virus [72]. However, more recent discussions about data sharing in subsequent epidemics and pandemics have focused on the need for rapidly sharing genome sequence data and not given weight to such concerns.

## Discussion

Powerful public, private and philanthropic stakeholders with an interest in advancing science and in promoting global health have accentuated the importance and merit of sharing health-related data rapidly in epidemics and pandemics. Data sharing in epidemics and pandemics is thought to further several values: utility, equity, solidarity, and reciprocity. Within the reviewed literature, utility and its associated norms were discussed substantially more than the remaining three values. Data sharing mandates and norms centered on utility have previously generated significant concerns related to equity.

Within the context of epidemics and pandemics, the equity-related norms discussed in the reviewed literature centered on capacity development, benefit sharing, equitable access to data, and researcher recognition. While these are consistent with the broader data sharing ethics literature, they are insufficient to promote equitable data sharing in epidemics and pandemics for reasons discussed in more detail below. First, the equity-related norms identified in this review advance several but not all dimensions of equity and social justice. Second, equity-related norms specific to data sharing are inadequate in themselves to address broader structural inequities in capacities to generate, share, and utilize health-related data in epidemics and pandemics.

### Dimensions of equity and social justice

Multiple dimensions of equity and social justice are described in the philosophical and ethics literatures: distributive, inclusion, recognition, power relations, and rights to self-development and adequate levels of well-being. Distributive accounts address the importance of fairly distributing burdens and benefits and reducing unfair disparities in the distribution of resources [74]. Inclusion encompasses a right to shape a decision-making space, to be present or represented (in diversity and numbers), to raise voice (spoken, written, or drawn), and be heard within that space [75–77]. It means ensuring all affected, including those whose voices are often less heard, are included in decision-making [78]. Scholars commonly equate fairness with *consensual* and *deliberative* decision-making [79–82], though some call for deliberations to be conflict-seeking [83]. Accounts of structural inequities highlight the importance of reducing unfair power relations such as subordination, exploitation, exclusion, violence, and colonality [78, 84, 85]. Recognition-based accounts call for addressing misrecognition in its various forms: (1) devaluing and stereotyping social groups, (2) rendering their knowledge, needs, and perspectives’ invisible (epistemic and cognitive injustice) and (3) failing to recognize group rights, including to sovereignty [78, 86–88]. Additional dimensions of equity and social justice address the importance of enabling self-development and human flourishing, including achieving an adequate level of wellbeing, for all, with debate ongoing as to whether that comprises a basic, sufficient, or optimal level of wellbeing [76, 78, 89–91]. Each of these dimensions is likely relevant to equitable data sharing. Aside from inclusion, they are all reflected in the norms identified in this study and the norms proposed to address concerns identified in this study. The alignment between the norms and dimensions of social justice and the relevance of inclusion to data sharing is discussed below.

The equity-related norms identified in this review seek to promote the well-being of those considered less advantaged (i.e., norms of capturing diversity, capacity development, fair benefits, equitable global access to benefits) and recognition (i.e., the norm of researcher recognition). Power inequalities were partially addressed through norms of national sovereignty and data stewardship. Largely missing, however, were data sharing norms that advance procedural fairness and inclusion. Procedural fairness considerations relate to how decisions are made, who makes them, and whose data are counted to develop solutions to combat epidemics and pandemics. Inclusion considerations relate to who participates, for what purpose, and how they participate. Inclusive approaches often call for involving a range of stakeholders at all levels of decision-making. Both are important from an equity perspective. They increase the likelihood data sharing will help alleviate suffering and reduces its chances of widening inequities. Merson et al. [92, p. 965] note that, in their experience, when the researchers responsible for data collection steer the design of secondary research, the resultant analyses are “relevant to the context where data are collected and maximize its potential to improve health.”

The equity-related concerns raised in the literature also suggest additional norms advancing recognition and distribution dimensions of social justice are needed for data sharing in epidemics and pandemics. The identified equity-related norms do not address concerns about the lack of non-biomedical research data being generated and shared and stigmatization. In effect, sharing biomedical and non-biomedical data may reinforce structural inequities and sharing biomedical research data and their benefits may be privileged. The latter means data sharing may not help address the systematic disadvantage created or worsened by an epidemic or pandemic as effectively as it could. Sharing non-biomedical research data can be critical to determine who is made vulnerable by an epidemic or pandemic and how they are made vulnerable and to effectively assess the impact of public health, economic, and other strategies to combat the epidemic or pandemic on those considered marginalized or disadvantaged. In addition, in the reviewed literature, benefit sharing norms were only discussed in the context of vaccines and biomedical research but should be broadened to encompass the benefits of non-biomedical research. Additional norms relating to cognitive justice (i.e., no knowledge system or type of data are privileged for sharing), and preventing harms are thus also needed for data sharing to advance equity in epidemics and pandemics. Such norms would advance the social justice dimensions of recognition and distribution by ensuring that harms are not disproportionately experienced by those already socially marginalized and by rendering different ways of knowing and types of inequity caused by epidemics and pandemics visible. More work is needed to articulate these norms (and norms of inclusion) comprehensively for data sharing, as it is beyond the scope of this paper to do so.

### Addressing structural inequities

As most recently demonstrated by the COVID-19 pandemic, epidemics and pandemics expose and exacerbate inequalities and systemic vulnerabilities, highlighting the importance of promoting social justice in global health responses [93]. Global health is, however, an inherently complex field of social, political, economic and scientific relationships in which various stakeholders have differing interests, priorities, and power [94]. This complex and inequitable landscape has prompted calls to ensure that approaches to scientific discovery and sharing the benefits and burdens of pandemic research are equitable and highlighted the importance of decolonising global health research [95–97].

Although international collaboration is widely encouraged during epidemics and pandemics, concerns regarding inequitable research collaborations during epidemics and pandemics were strongly voiced in the literature identified by this study [25, 27, 42, 48]. Both North–South and South–South collaborations during epidemics and pandemics may reflect asymmetries in the division of labor and decision-making, and comprise new relations of “data colonialism” [25]. During the Zika epidemic, for example, LMIC partners voiced concerns about being excluded from setting research objectives and designing studies, resulting in being relegated to operational roles and restricting their capacity to develop the skills needed to originate and conduct research addressing regional priorities [48]. Concerns have also been expressed that asymmetries in capacity, infrastructure, and technology reinforce a division of labor where LMIC partners collect data and HIC partners analyze them, and data analysis is more valued by the research enterprise than data collection [25, 48]. To effectively promote equitable data sharing in epidemics and pandemics, it is thus important not just to develop equity-related norms for such sharing, but also to address the structural inequalities in the global health landscape from which research questions are prioritized and data are collected, analyzed, and shared. Equity-related norms specific to data sharing are inadequate in themselves to rectify these broader structural inequities and a global health enterprise-wide approach (rather than data sharing-specific approach) is needed.

### Ways forward

In the short-term, it is important to draw on dimensions of social justice to supplement existing norms for data sharing in epidemics and pandemics to support equitable practice. Work is needed to further explicate data sharing norms in relation to inclusion, preventing harms, and cognitive justice. In addition, work must be done to articulate how tensions between equity and utility norms should be navigated when sharing data during epidemics and pandemics. This study found that the utility-related norm of rapid, real-time data sharing is in tension with equity-related norms of researcher recognition and national sovereignty. Beyond this, we suggest that other tensions between equity and utility may emerge. Capacity development and achieving inclusion and fair participatory processes can be time-intensive, whereas utility-related norms emphasize speed. Minimizing harms may not be a fast process either. Slowing down to achieve capacity development, harm mitigation, and inclusive decision-making processes about data access will be in tension with rapid, real-time sharing norms. Equitable access could entail giving priority access to research data to LMIC researchers and source country researchers, which would likely also hamper rapid sharing. Future ethics research should explore how to balance the tensions identified here, potentially in a way that gives more weight to utility than in non-epidemic contexts given the priority placed on promoting utility in epidemic contexts.

In particular, in non-epidemic contexts, multiple protection measures to ensure LMIC researchers’ recognition and access to data have been proposed. Examples include acknowledgement and authorship requirements, and extended embargos on the release of data for secondary analysis which privilege LMIC access. (Table 2). We posit that, while recognition requirements encompassing acknowledgment and authorship should be upheld in epidemics and pandemics, mechanisms that restrict data access are more difficult to justify, given the role that such access could play in accelerating effective public health responses. We further suggest that substantive amendments to equity promoting processes such as capacity strengthening and procedural fairness would require careful consideration if seeking to avoid approaches to data sharing that exacerbate existing inequalities. Future research should explore whether such amendments are ethically justified and, if so, what they might look like.

Immediate tasks thus comprise work to articulate additional equity norms for data sharing in epidemics and pandemics and to determine how tensions between them and utility norms should be navigated. It is important that data sharing norms in epidemics and pandemics do not exacerbate existing inequalities, nor adversely affect the capacities of countries to effectively respond to public health emergencies. In the longer term, it is critical to address structural inequities in the global health landscape, which will further help reduce tensions between utility and equity. There is substantial thinking and work going on about how to do this and how to decolonize global health in practice, and some initial tools and guidance on achieving fairness in research initiatives have been developed [21, 98].

## Limitations of the study

The amount of literature related to COVID-19 is expanding at an exceptionally rapid rate. Yet, literature focusing on norms and values related to pandemic data sharing was relatively limited. It nonetheless provided sufficient data to address our research questions. This study gathered articles, editorials, blogs, news articles, reports, and guidance and policy documents related to data sharing in epidemics and pandemics prior to July 2020. Literature published since then has not been systematically reviewed to inform this paper. The study was also limited to English language articles.

Although double screening of all materials is desirable in scoping reviews, it was not implemented in this case due to the volume of potential references identified. The full text of all 197 resources from the Epidemic Ethics Database were screened because many identified documents, such as editorials and policy statements, came without abstracts. To minimize error and bias, 15% of full text papers were co-reviewed.

## Conclusions

This study found support for equity being advanced by data sharing in epidemics and pandemics, however utility-related norms were prioritized over equity. Norms for equitable data sharing in epidemics and pandemics were identified but require further development, particularly in relation to inclusion, preventing harms, and cognitive justice. Addressing structural inequities in the wider global health landscape is also needed to achieve equitable data sharing in epidemics and pandemics. Several equity-related norms are in tension with utility-related norms. Further investigation into approaches to promote equity while giving appropriate weight to utility maximization is needed. We hope that our findings spark more dialogue and ethics research to investigate these matters in order to further develop an account of equitable data sharing in epidemics and pandemics.

## Supplementary Information

**Additional file 1**: Search strategies. Full search strategies for formal literature search of Embase, Medline, Public Health, and Web of Science.

**Additional file 2**: Included literature on data sharing in epidemics and pandemics. Full list of articles included from the literature review.

## Data Availability

The datasets used and/or analysed during the current study are available from the corresponding author on reasonable request.

## Abbreviations

HIC: High-income country
LMIC: Low- and middle-income country

## Footnotes

1 Knowledge and benefit sharing refer to sharing the information generated by interpreting raw datasets (e.g., sharing primary or secondary research outputs) and any products or other benefits that are generated from that information.

2 We recognize that the matter of knowledge and benefit sharing has been the focus of substantial discussion and debate over many years in the academic literature and has been included in several international agreements (e.g., UNESCO Bioethics Declaration, Nagoya Protocol). We do not address such a large topic in this paper beyond describing how it is discussed in the epidemic and pandemic data sharing literature.

## References

[1] Yasinski E (2020). Scientists scrutinize new Coronavirus genome for answers.

[2] Global Research Collaboration for Infectious Disease Preparedness (GLOPID-R). Principles of data sharing in public health emergencies. 2018.

[3] Moorthy V, Restrepo AMH, Preziosi M, Swaminathan S (2020). Data sharing for novel coronavirus (COVID-19). Bull World Health Organ.

[4] Anane-Sarpong E, Wangmo T, Ward CL, Sankoh O, Tanner M, Elger BS (2018). “You cannot collect data using your own resources and put It on open access”: perspectives from Africa about public health data-sharing. Dev World Bioeth.

[5] Bezuidenhout L, Chakauya E (2018). Hidden concerns of sharing research data by low/middle-income country scientists. Glob Bioeth.

[6] Serwadda D, Ndebele P, Grabowski MK, Bajunirwe F, Wanyenze RK (2018). Open data sharing and the Global South—Who benefits?. Science.

[7] Barnes KI, Canario JA, Vernekar SS, Goudar SS, Espinal R, Merson L (2019). Equitable data sharing: challenges and suggestions for ways forward. Wellcome Open Res.

[8] Birney E, Hudson TJ, Green ED, Gunter C, Eddy S, Rogers J (2009). Prepublication data sharing. Nature.

[9] Pisani E, Aaby P, Breugelmans JG, Carr D, Groves T, Helinski M (2016). Beyond open data: realising the health benefits of sharing data. BMJ.

[10] Bull S, Roberts N, Parker M (2015). Views of ethical best practices in sharing individual-level data from medical and public health research: a systematic scoping review. JERHRE.

[11] Merson L, Gaye O, Guerin PJ (2016). Avoiding data dumpsters—toward equitable and useful data sharing. N Engl J Med.

[12] Arksey H, O'Malley L (2005). Scoping studies: towards a methodological framework. Int J Soc Res Methodol.

[13] Armstrong R, Hall BJ, Doyle J, Waters E (2011). ‘Scoping the scope’ of a cochrane review. J Public Health.

[14] Braun V, Clarke V (2006). Using thematic analysis in psychology. Qual Res Psychol.

[15] Krieger N. COVID-19, data, and health justice. 2020. Available from: https://www.commonwealthfund.org/blog/2020/covid-19-data-and-health-justice.

[16] Morten CJ, Kapczynski A, Krumholz HM, Ross JS (2020). To help develop the safest, most effective Coronavirus tests, treatments, and vaccine, ensure public access to clinical research data. Health Aff.

[17] Presidential Commission for the Study of Bioethical Issues. Ethics and Ebola: public health planning and response. 2015. Washington DC: Presidential Commission for the Study of Bioethical Issues.

[18] World Heath Organization. Ethical considerations in developing a public health response to pandemic influenza. 2007. Geneva: World Health Organization.

[19] Langat P, Pisartchik D, Silva D, Bernard C, Olsen K, Smith M (2011). Is There a duty to share? Ethics of sharing research data in the context of public health emergencies. Public Health Ethics.

[20] McLennan S, Celi LA, Buyx A (2020). COVID-19: Putting the general data protection regulation to the test. JMIR Public Health Surveill.

[21] Nuffield Council on Bioethics. Research in global health emergencies: ethical issues. 2019. London: Nuffield Council on Bioethics.

[22] Anonumous (2015). Sharing data to save lives. Nat Med.

[23] Anonymous (2016). Don't wait to share data on Zika. Nat Microbiol.

[24] Gewin V (2020). Six tips for data sharing in the age of the coronavirus. Nature.

[25] Modjarrad K, Moorthy VS, Millett P, Gsell PS, Roth C, Kieny MP (2016). Developing global norms for sharing data and results during public health emergencies. PLoS Med.

[26] Enserink M (2006). Avian influenza—AsH5N1 keeps spreading, a call to release more data. Science.

[27] Jorge VD, Albagli S. Research data sharing during the Zika virus public health emergency. Inf Res. 2020;25(1).

[28] Armstrong S (2020). Covid-19: Deadline for roll out of UK's tracing app will be missed. BMJ.

[29] Bogner P, Capua I, Cox NJ, Lipman DJ (2006). A global initiative on sharing avian flu data. Nature.

[30] Brimacombe KR, Zhao T, Eastman RT, Hu X, Wang K, Backus M (2020). An OpenData portal to share COVID-19 drug repurposing data in real time. BioRxivthe.

[31] Cai Q, Mi Y, Chu Z, Zheng Y, Chen F, Liu Y (2020). Demand analysis and management suggestion: sharing epidemiological data among medical institutions in megacities for epidemic prevention and control. J Shanghai Jiaotong Univ Sci.

[32] Cheng CKY, Lau EHY, Ip DKM, Yeung ASY, Ho LM, Cowling BJ (2009). A profile of the online dissemination of national influenza surveillance data. BMC Pub Health.

[33] Cherif MS, Craig E, Strudwick S, Hawryszkiewycz A, Merson L (2018). The Ebola Data Platform: a novel collaboration for training and research in emerging infections. Am J Trop Med Hyg.

[34] Cosgriff CV, Ebner DK, Celi LA (2020). Data sharing in the era of COVID-19. Lancet Digit Health.

[35] Enserink M (2006). Avian influenza—pushed by an outsider, scientists call for global plan to share flu data. Science.

[36] He Y, Yu H, Ong E, Wang Y, Liu Y, Huffman A (2020). CIDO, a community-based ontology for coronavirus disease knowledge and data integration, sharing, and analysis. Sci Data.

[37] Salzberg S, Ghedin E, Spiro D (2006). Shared data are key to beating threat from flu. Nature.

[38] Shu YL, McCauley J (2017). GISAID: Global initiative on sharing all influenza data—from vision to reality. Euro Surveill.

[39] Vong S, O'Leary M, Feng ZJ (2014). Early response to the emergence of influenza A(H7N9) virus in humans in China: the central role of prompt information sharing and public communication. Bull World Health Organ.

[40] World Health Organization. Guidance for managing ethical issues in infectious disease outbreaks. 2016.

[41] Pisani E, Ghataure A, Merson L. Data sharing in public health emergencies: a study of current policies, practices and infrastructure supporting the sharing of data to prevent and respond to epidemic and pandemic threats. 2018. Wellcome Trust J Contrib. 10.6084/M9.FIGSHARE.5897608.V1.

[42] da Costa MP, Leite FCL (2019). Factors influencing research data communication on Zika virus: a grounded theory. J Doc.

[43] World Heath Organization. Pandemic influenza preparedness (PIP) framework. 2011. Geneva: World Health Organization.

[44] African Academy of Sciences (2020). Statement of the African Academy of Sciences’ Biospecimens and Data Governance Committee on COVID-19: ethics, governance and community engagement in times of crises.

[45] Bayram M, Springer S, Garvey CK, Ozdemir V (2020). COVID-19 digital health innovation policy: a portal to alternative futures in the making. OMICS.

[46] Gorina Y, Redd JT, Hersey S, Jambai A, Meyer P, Kamara AS (2020). Ensuring ethical data access: the Sierra Leone Ebola Database (SLED) model. Ann Epidemiol.

[47] Aubusson K (2020). Global data-sharing alliance key to finding COVID-19 cures fast.

[48] Guzman JAC, Espinal R, Baez J, Melgen RE, Rosario PAP, Mendoza ER (2017). Ethical challenges for international collaborative research partnerships in the context of the Zika outbreak in the Dominican Republic: a qualitative case study. Health Res Policy Syst.

[49] GISAID EpiFlu™ Database Access Agreement [press release]. 2011.

[50] Lawson C, Rourke M (2016). Open access DNA, RNA and amino acid sequences: the consequences and solutions for the international regulation of access and benefit sharing. J Law Med.

[51] McNutt M (2016). Data sharing. Science.

[52] Liu Y, Salwi S, Drolet BC (2020). Multivalue ethical framework for fair global allocation of a COVID-19 vaccine. J Med Ethics.

[53] Rahimi F, Talebi Bezmin Abadi A (2020). Transparency and information sharing could help abate the COVID-19 pandemic. Infect Control Hosp Epidemiol.

[54] Song P, Karako T (2020). COVID-19: Real-time dissemination of scientific information to fight a public health emergency of international concern. Biosci Trends.

[55] Whitty CJ, Mundel T, Farrar J, Heymann DL, Davies SC, Walport MJ (2015). Providing incentives to share data early in health emergencies: the role of journal editors. Lancet.

[56] Chretien JP, Rivers CM, Johansson MA (2016). Make data sharing routine to prepare for public health emergencies. PLoS Med.

[57] Research Fairness Initiative. RFI summary guide. 2018. Available from: https://rfi.cohred.org/wp-content/uploads/RFI_Summary_Guide_1.pdf.

[58] COVID-19 Clinical Research Coalition (2020). Global coalition to accelerate COVID-19 clinical research in resource-limited settings. Lancet.

[59] Kallas EG, O'Connor DH (2016). Real-time sharing of Zika virus data in an Interconnected World. JAMA Pediatr.

[60] Kmietowicz Z (2016). Research bodies vow to share data on Zika. BMJ.

[61] Berry I, Soucy JPR, Tuite A, Fisman D, Data C-CO (2020). Open access epidemiologic data and an interactive dashboard to monitor the COVID-19 outbreak in Canada. CMAJ.

[62] Crowcroft NS, Rosella LC, Pakes BN (2014). The ethics of sharing preliminary research findings during public health emergencies: a case study from the 2009 influenza pandemic. Euro Surveill.

[63] Lawpoolsri S, Kaewkungwal J, Khamsiriwatchara A, Sovann L, Sreng B, Phommasack B (2018). Data quality and timeliness of outbreak reporting system among countries in Greater Mekong subregion: challenges for international data sharing. PLoS Negl Trop Dis.

[64] Wise J (2020). Data transparency: "nothing has changed since Tamiflu". BMJ.

[65] Worby CJ, Lipsitch M, Hanage WP (2017). Shared genomic variants: identification of transmission routes using pathogen deep-sequence data. Am J Epidemiol.

[66] Yozwiak NL, Schaffer SF, Sabeti PC (2015). Data sharing - make outbreak research open access. Nature.

[67] Abramowitz S. G-VT, Webb J., Tappan J., Uretsky E., Varanda-Ferreira J., Mason K., Beyer M., Collin C., & Sall A. Data sharing in public health emergencies: anthropological and historical perspectives on data sharing during the 2014–2016 Ebola epidemic and the 2016 Yellow Fever epidemic. 2018.

[68] Georgetown University Center for Global Health Science and Security. MERS-CoV data sharing case study report. 2018.

[69] Goldacre B, Harrison S, Mahtani KR, Heneghen C. WHO consultation on data and results sharing during public health emergencies. 2015.

[70] Heyerdahl L, Njanpop-Lafourcade B, Sauvageot D, Delrieu I, Thioune A, Guillermet E. Data sharing: a cholera case study. Final report. 2018.

[71] Calain P, Fiore N, Poncin M, Hurst SA (2009). Research ethics and international epidemic response: the case of Ebola and Marburg Hemorrhagic Fevers. Public Health Ethics.

[72] Hurlbut JB (2017). A science that knows no country: pandemic preparedness, global risk, sovereign science. Big Data Soc.

[73] van Roode M, dos Santos Ribeiro C, Farag E, Ahmed M, Moustafa A, van de Burgwal L, et al. Data sharing in public health emergencies: analysis of barriers and enablers from an outbreak response perspective (SHARE). 2018. London: Wellcome Trust, GLOPID-R, UK Department of International Development.

[74] Macklin R (2005). Double standards in medical research in developing countries.

[75] Cornwall A, Cornwall A (2011). Whose voices? Whose choices? Reflections on gender and participatory development. The participation reader.

[76] Young IM (2000). Inclusion and democracy.

[77] Crocker DA (2008). Ethics of global development: agency, capability, and deliberative democracy.

[78] Young IM (1990). Justice and the politics of difference.

[79] Chemhuru M (2017). Gleaning the social contract theory from African communitarian philosophy. S Afr J Philos.

[80] Letseka M (2014). Ubuntu and justice as fairness. Mediterr J Soc Sci.

[81] Gutmann A, Thompson D (2004). Why deliberative democracy? Princeton.

[82] Daniels N (2008). Just health: meeting health needs fairly.

[83] Mouffe C, Benhabib S (1996). Democracy, power, and the “political". Democracy and difference: contesting the boundaries of the political.

[84] Powers M, Faden R (2019). Structural injustice: power, advantage, and human rights.

[85] Maldonado-Torres N (2007). On the colonality of being. Cult Stud.

[86] Fraser N (1997). Justice interruptus critical reflections on the "postsocialist" condition.

[87] Fricker M (2007). Epistemic injustice: power and the ethics of knowing.

[88] Santos B (2014). Epistemologies of the South: justice against epistemicide.

[89] Powers M, Faden R (2006). Social justice: the moral foundations of public health and health policy.

[90] Nussbaum M (2000). Women and human development: the capabilities approach.

[91] Venkatapuram S (2011). Health justice: an argument from the capabilities approach.

[92] Merson L, Guérin PJ, Barnes KI, Ntoumi F, Gaye O (2018). Secondary analysis and participation of those at the data source. Lancet Glob Health.

[93] Ivers LC, Walton DA (2020). COVID-19: global health equity in pandemic response. Am J Trop Med Hyg.

[94] Shiffman J (2015). Global health as a field of power relations: a response to recent commentaries. Int J Health Policy Manag.

[95] Coleman CH (2020). Equitably sharing the benefits and burdens of research: Covid-19 raises the stakes. Ethics Hum Res.

[96] Kavanagh MM, Erondu NA, Tomori O, Dzau VJ, Okiro EA, Maleche A (2020). Access to lifesaving medical resources for African countries: COVID-19 testing and response, ethics, and politics. Lancet.

[97] Atuire C, Bull S. (in press) COVID-19 heightens the imperative to decolonize global health research. Global Justice.

[98] Anonymous (2016). Publishers and funders push sharing of Zika data. Chem Eng News.

