# Supplementary material for: Equitable data sharing in epidemics and pandemics
Source: BMC Med Ethics. 2021 Oct 6;22:136. doi: 10.1186/s12910-021-00701-8 (PMC8493940; doi:10.1186/s12910-021-00701-8)
Supplement: Supplementary file 1 — Additional file 1: Search strategies. Full search strategies for formal literature search of Embase, Medline, Public Health, and Web of Science. [file 12910_2021_701_MOESM1_ESM.docx]

**Additional File 1: Search Strategies**

**Public Health, 2 July 2020**

(title:("Severe Acute Respiratory Syndrome" or SARS or "Middle East Respiratory Syndrome" or MERS or Zika or Ebola or influenza or flu or covid or COVID-19 or coronavirus or "corona virus") OR ab:("Severe Acute Respiratory Syndrome" or SARS or "Middle East Respiratory Syndrome" or MERS or Zika or Ebola or influenza or flu or covid or COVID-19 or coronavirus or "corona virus")) AND (title:((data or database* or dataset*) and (share* or sharing or release* or releasing or disseminat* or distribut* or export* or recycl* or access* or reuse*)) OR title:(information and (share* or sharing or release* or releasing or disseminat* or distribut* or export* or recycl* or access* or reuse*)) OR title:(finding* and (share* or sharing or release* or releasing or disseminat* or distribut* or export* or recycl* or access* or reuse*)) OR title:(result* and (share* or sharing or release* or releasing or disseminat* or distribut* or export* or recycl* or access* or reuse*)) OR title:(data and research)) yr:[2010 TO 2020]

**Medline Ovid, 2 July 2020**

1. ((data or database* or dataset*) and (share* or sharing or release* or releasing or disseminat* or distribut* or export* or recycl* or access* or reuse*)).ti.

2. (information and (share* or sharing or release* or releasing or disseminat* or distribut* or export* or recycl* or access* or reuse*)).ti.

3. (finding* and (share* or sharing or release* or releasing or disseminat* or distribut* or export* or recycl* or access* or reuse*)).ti.

4. (result* and (share* or sharing or release* or releasing or disseminat* or distribut* or export* or recycl* or access* or reuse*)).ti.

5. (data and research).ti.

6. ((data or database*) adj5 open access).ti,ab.

7. (tracking app* or tracing app* or mobile phone* or COVID app* or immunity passport*).ti,ab.

8. 1 or 2 or 3 or 4 or 5 or 6 or 7

9. (Severe Acute Respiratory Syndrome or SARS or Middle East Respiratory Syndrome or MERS or Zika or Ebola or influenza or flu or covid or COVID-19 or coronavirus or corona virus).ti,ab.

10. Severe Acute Respiratory Syndrome/ep, pc or Middle East Respiratory Syndrome Coronavirus/ep, pc or Zika Virus/ep, pc or Hemorrhagic Fever, Ebola/ep, pc or Influenza, Human/ep, pc or Influenza A Virus, H1N1 Subtype/ep, pc or Influenza A Virus, H5N1 Subtype/ep, pc or Influenza A Virus, H3N2 Subtype/ep, pc or Coronavirus Infections/ep, pc

11. 9 or 10

12. 8 and 11

**EMBASE Ovid, 2 July 2020**

1. ((data or database* or dataset*) and (share* or sharing or release* or releasing or disseminat* or distribut* or export* or recycl* or access* or reuse*)).ti.

2. (information and (share* or sharing or release* or releasing or disseminat* or distribut* or export* or recycl* or access* or reuse*)).ti.

3. (finding* and (share* or sharing or release* or releasing or disseminat* or distribut* or export* or recycl* or access* or reuse*)).ti.

4. (result* and (share* or sharing or release* or releasing or disseminat* or distribut* or export* or recycl* or access* or reuse*)).ti.

5. (data and research).ti.

6. ((data or database*) adj5 open access).ti,ab.

7. (tracking app* or tracing app* or mobile phone* or COVID app* or immunity passport*).ti,ab.

8. 1 or 2 or 3 or 4 or 5 or 6 or 7

9. (Severe Acute Respiratory Syndrome or SARS or Middle East Respiratory Syndrome or MERS or Zika or Ebola or influenza or flu or covid or COVID-19 or coronavirus or corona virus).ti,ab.

10. severe acute respiratory syndrome/ep, pc or Middle East respiratory syndrome coronavirus/ep, pc or Zika virus/ep, pc or Ebola hemorrhagic fever/ep, pc or influenza/ep, pc or "Influenza A virus (H1N1)"/ or "Influenza A virus (H3N2)"/ or Coronavirus infection/ep, pc

11. 9 or 10

12. 8 and 11

13. limit 12 to (article or article in press)

**Web of Science, 2 July 2020**

1. ti=((data or database* or dataset*) and (share* or sharing or release* or releasing or disseminat* or distribut* or export* or recycl* or access* or reuse*) )
2. ti=(information and (share* or sharing or release* or releasing or disseminat* or distribut* or export* or recycl* or access* or reuse*) )
3. ti=(finding* and (share* or sharing or release* or releasing or disseminat* or distribut* or export* or recycl* or access* or reuse*) )
4. ti=(result* and (share* or sharing or release* or releasing or disseminat* or distribut* or export* or recycl* or access* or reuse*) )
5. ti=(data and research)
6. ts=((data or database*) and "open access")
7. ts=("tracking app*" or "COVID app*" or "immunity passport*")
8. #7 OR #6 OR #5 OR #4 OR #3 OR #2 OR #1
9. ts=("Severe Acute Respiratory Syndrome" or SARS or "Middle East* Respiratory Syndrome" or MERS or Zika or Ebola or influenza or flu or covid or COVID-19 or coronavirus or "corona virus")
10. #9 AND #8
