# Supplementary material for: Equitable data sharing in epidemics and pandemics
Source: BMC Med Ethics. 2021 Oct 6;22:136. doi: 10.1186/s12910-021-00701-8 (PMC8493940; doi:10.1186/s12910-021-00701-8)
Supplement: Supplementary file 2 — Additional file 2: Included literature on data sharing in epidemics and pandemics. Full list of articles included from the literature review. [file 12910_2021_701_MOESM2_ESM.docx]

**Additional File 2**

**Included literature on data sharing in epidemics and pandemics**

|  | **Authors** | **Year** | **Title** | **Type of Article** | **Type of Data Sharing Discussed** |
| --- | --- | --- | --- | --- | --- |
| 1 | Abramowitz S, Giles-Vernick T, Webb J, Tappan J, Uretsky E, Varanda-Ferreira J, Mason K, Beyer M, Collin C, Sall A | 2018 | Data sharing in public health emergencies: anthropological and historical perspectives on data sharing during the 2014-2016 Ebola epidemic and the 2016 Yellow Fever epidemic | Report | Epidemiology, Research, and Viral genomic |
| 2 | African Academy of Sciences. | 2020 | Statement of the African Academy of Sciences’ Biospecimens and Data Governance Committee on COVID-19: ethics, governance and community engagement in times of crises | Statement | Epidemiology, Viral genomic |
| 3 | Amaro RE, Mulholland AJ. | 2020 | A community letter regarding sharing biomolecular simulation data for COVID-19 | Letter | Research (Modelling) |
| 4 | Anonymous | 2016 | Don't wait to share data on Zika | Editorial | Epidemiology and Clinical |
| 5 | Anonymous | 2016 | Publishers and funders push sharing of Zika data | Editorial | Research |
| 6 | Anonymous | 2015 | Sharing data to save lives | Editorial | Epidemiology, Clinical, and Viral genomic |
| 7 | Aubusson,K. | 2020 | Global data-sharing alliance key to finding COVID-19 cures fast | News | Research |
| 8 | Berry I, Soucy JPR, Tuite A, Fisman D, Data C-CO. | 2020 | Open access epidemiologic data and an interactive dashboard to monitor the COVID-19 outbreak in Canada | Letter | Epidemiology |
| 9 | Blomberg N,  Lauer KB. | 2020 | Connecting data, tools and people across Europe: ELIXIR's response to the COVID-19 pandemic | Article | Epidemiology, Clinical, Research, and Viral genomic |
| 10 | Bogner P, Capua I, Cox NJ, Lipman DJ. | 2006 | A global initiative on sharing avian flu data. Nature | Letter | Epidemiology and Viral genomic |
| 11 | Brimacombe KR, Zhao T, Eastman RT, Hu X, Wang K, Backus M, Baljinnyam B., Chen C. Z., Chen L., Eicher T, Ferrer M, Fu Y, Gorshkov K, Guo H, Hanson QM, Itkin Z, Kales SC, Klumpp-Thomas C, Lee EM, Michael S, Mierzwa T, Patt A, Pradhan M, Renn A, Shinn P, Shrimp JH, Viraktamath A, Wilson KM, Xu M, Zakharov AV, Zhu W, Zheng W, Simeonov A, Mathé, EA, Lo DC, Hall MD, Shen, M. | 2020 | An OpenData portal to share COVID-19 drug repurposing data in real time | Article | Research |
| 12 | Cai Q, Mi Y, Chu Z, Zheng Y, Chen F, Liu Y | 2020 | Demand analysis and management suggestion: sharing epidemiological data among medical institutions in megacities for epidemic prevention and control | Feature | Epidemiology |
| 13 | Calain P, Fiore N, Poncin M, Hurst, SA. | 2009 | Research ethics and international epidemic response: the case of Ebola and Marburg Hemorrhagic Fevers | Article | Epidemiology, Clinical, Viral genomic |
| 14 | Cheng CKY, Lau EHY, Ip DKM, Yeung ASY, Ho LM, Cowling BJ. | 2009 | A profile of the online dissemination of national influenza surveillance data | Article | Epidemiology |
| 15 | Cherif MS, Craig E, Strudwick S, Hawryszkiewycz A, Merson L. | 2018 | The Ebola Data Platform: a novel collaboration for training and research in emerging infections | Conference abstract | Epidemiology and Clinical |
| 16 | Chretien JP, Rivers CM, Johansson MA. | 2016 | Make data sharing routine to prepare for public health emergencies | Article | Research |
| 17 | Cosgriff CV, Ebner DK, Celi LA. | 2020 | Data sharing in the era of COVID-19 | Letter | Epidemiology for research purposes |
| 18 | COVID-19 Clinical Research Coalition. | 2020 | Global coalition to accelerate COVID-19 clinical research in resource-limited settings | Article | Research |
| 19 | Crowcroft NS, Rosella LC, Pakes BN. | 2014 | The ethics of sharing preliminary research findings during public health emergencies: a case study from the 2009 influenza pandemic | Article | Research |
| 20 | da Costa MP, Leite FCL. | 2019 | Factors influencing research data communication on Zika virus: a grounded theory | Article | Research |
| 21 | Dye C, Bartolomeos K, Moorthy V, Kieny MP | 2016 | Data sharing in public health emergencies:  a call to researchers | Editorial | Research |
| 22 | Dyer C. | 2020 | Covid-19: rules on sharing confidential patient information are relaxed in England | News | Epidemiology |
| 23 | Enserink M. | 2006 | Avian influenza - pushed by an outsider, scientists call for global plan to share flu data | News | Viral genomic |
| 24 | Enserink M. | 2006 | Avian influenza - AsH5N1 keeps spreading, a call to release more data | News | Research |
| 25 | Evans NG, Hills K, and Levine AC. | 2020 | How should the WHO guide access and benefit sharing during infectious disease outbreaks? | Article | Viral genomic |
| 26 | Georgetown University Center for Global Health Science and Security | 2018 | Data sharing during the West Africa Ebola public health emergency:  case study report | Report | Epidemiology, Clinical, Research, and Viral genomic |
| 27 | Georgetown University Center for Global Health Science and Security | 2018 | MERS-CoV data sharing case study report | Report | Epidemiology, Clinical, Research, and Viral genomic |
| 28 | Gewin V. | 2020 | Six tips for data sharing in the age of the coronavirus | Feature | Research |
| 29 | GISAID | 2011 | Database access agreement | Statement  https://www.gisaid.org/registration/terms-of-use/ | Viral Genomic |
| 30 | GLOPID-R | 2018 | Principles of data sharing in public health emergencies | Guidance and Policy | Research |
| 31 | GLOPID-R | 2017 | Roadmap for data sharing in public health emergencies | Report | Research |
| 32 | Goldacre B, Harrison S, Mahtani KR, Heneghan C. | 2015 | WHO consultation on data and results sharing during public health emergencies | Report | Epidemiology, Clinical, Research, and Viral genomic |
| 33 | Gorina Y, Redd JT, Hersey S, Jambai A, Meyer P, Kamara AS, Kamara A, Harding JD, Bangura B, Kamara MAM. | 2020 | Ensuring ethical data access: the Sierra Leone Ebola Database (SLED) model | Article | Research |
| 34 | Guzman JAC, Espinal R, Baez J, Melgen RE, Rosario PAP, Mendoza ER. | 2017 | Ethical challenges for international collaborative research partnerships in the context of the Zika outbreak in the Dominican Republic: a qualitative case study | Article | Research |
| 35 | He Y, Yu H, Ong E, Wang Y, Liu Y, Huffman A, Hsin-hui H, Beverly J, Hur J, Yang X, Chen L, Omenn GS, Athey B, Smith B. | 2020 | CIDO, a community-based ontology for coronavirus disease knowledge and data integration, sharing, and analysis | Article | Research |
| 36 | Heyerdahl, L, Njanpop-Lafourcade BM, Sauvageot D, Delrieu I, Thioune A, Guillermet E. | 2018 | Data sharing: a cholera case study | Report | Epidemiology, Clinical, Research, and Viral genomic |
| 37 | Holmes BJ, Henrich N, Hancock S, Lestou V. | 2009 | Communicating with the public during health crises: experts' experiences and opinions | Article | Doesn’t specify beyond data |
| 38 | Holub, P, Kozera, L, Florindi, F, van Enckevort E, Swertz M, Reihs R, Wutte A, Valík D, Mayrhofer, MT on behalf of the BBMRI-ERIC community. | 2020 | BBMRI-ERIC’s contributions to research and knowledge exchange on COVID-19 | Article | Research |
| 39 | Hurlbut, JB | 2017 | A science that knows no country: pandemic preparedness, global risk,  sovereign science | Article | Viral genomic |
| 40 | Jorge VD, Albagli S. | 2020 | Research data sharing during the Zika virus public health emergency | Article | Research |
| 41 | Kallas EG, O'Connor DH. | 2016 | Real-Time sharing of zika virus data in an interconnected world | Commentary | Research |
| 42 | Katz R, Standley CJ, Kornblet S, Sorrell E, Vaught A, Fischer JE. | 2017 | Information-sharing and disease reporting in a new era of international frameworks and communication technology: Middle East Respiratory Syndrome Coronavirus and Ebola Virus disease outbreaks | Chapter | Epidemiology |
| 43 | Kim AHJ, Sparks JA, Liew JW, Putman MS, Berenbaum F, Duarte-García A, Graef ER, Korsten P, Sattui SE, Sirotich E, Ugarte-Gil MF, Webb K, Grainger R. | 2020 | Rush to judgment? rapid reporting and dissemination of results and Its consequences regarding the use of Hydroxychloroquine for COVID-19 | Article | Research |
| 44 | Kmietowicz Z. | 2016 | Research bodies vow to share data on Zika | News | Research |
| 45 | Krieger, N. | 2020 | COVID-19, data, and health justice | Blog | Epidemiology, Clinical |
| 46 | Langat, P., Pisartchik, D., Silva, D., Bernard C, Olsen K, Smith M, Sahni S, Upshur R. | 2011 | Is there a duty to share? ethics of sharing research data in the context of public health emergencies | Article | Research |
| 47 | Lawpoolsri S, Kaewkungwal J, Khamsiriwatchara A, Sovann L, Sreng B, Phommasack B, Kitthiphong V, Nyein SL, Myint NW, Vung ND, Hung P, Smolinski MS, Crawley AW, Oo MK. | 2018 | Data quality and timeliness of outbreak reporting system among countries in Greater Mekong subregion: challenges for international data sharing | Article | Epidemiology |
| 48 | Lawson C, Rourke M. | 2016 | Open access DNA, RNA and amino acid sequences: the consequences and solutions for the international regulation of access and benefit sharing | Article | Viral genomic |
| 49 | Liverani M, Teng S, Le MS, Coker R. | 2018 | Sharing public health data and information across borders: lessons from Southeast Asia | Article | Epidemiology |
| 50 | McNutt M. | 2016 | Data sharing | Editorial | Research |
| 51 | Modjarrad K, Moorthy VS, Millett P, Gsell PS,  Roth C, Kieny M-P. | 2016 | Developing Global  Norms for Sharing Data and Results during Public  Health Emergencies. | Article | Epidemiology, Clinical, Research, and Viral genomic |
| 52 | Moorthy, V, Henao Restrepo, AM, Preziosi MP, Swaminathan, S. | 2020 | Data sharing for novel coronavirus (COVID-19). | Article | Research |
| 53 | Morten CJ, Kapczynski A, Krumholz HM, Ross JS. | 2020 | To help develop the safest, most effective Coronavirus tests, treatments, and vaccine, ensure public access to clinical research data | Blog | Research |
| 54 | Nuffield Council | 2019 | Research in global health emergencies: ethical issues | Guidance and Policy | Research |
| 55 | Olson DR, Paladini M, Lober WB, Buckeridge DL. | 2011 | Applying a new model for sharing population health data to national syndromic influenza surveillance: DiSTRIBuTE project proof of concept, 2006 to 2009 | Article | Epidemiology |
| 56 | Pisani E, Ghataure A, Merson L. | 2018 | Data sharing in public health emergencies | Report | Epidemiology, Clinical, Research, and Viral genomic |
| 57 | Presidential Commission for the Study of Bioethical Issues. | 2015 | Ethics and Ebola: public health planning and response | Guidance and Policy | Viral genomic |
| 58 | Rahimi F, Abadi ATB. | 2020 | Ethical and sensible dissemination of information during the COVID-19 pandemic | Letter | Research |
| 59 | Rogers WA, Street JM, Braunack-Mayer AJ, Hiller JE, FluViews T. | 2009 | Pandemic influenza communication: views from a deliberative forum | Article | Epidemiology |
| 60 | Salzberg S, Ghedin E, Spiro D. | 2006 | Shared data are key to beating threat from flu | Letter | Research |
| 61 | Shamoo AE. | 2020 | Validate the integrity of research data on COVID 19 | Editorial | Research |
| 62 | Shu YL, McCauley J. | 2017 | GISAID: global initiative on sharing all influenza data - from vision to reality | Editorial | Viral genomic |
| 63 | Song P, Karako T. | 2020 | COVID-19: real-time dissemination of scientific information to fight a public health emergency of international concern | Editorial | Research |
| 64 | Tangcharoensathien V,  Boonperm J, Jongudomsuk P. | 2010 | Sharing health data: developing country perspectives | Article | Research |
| 65 | TWAS | 2020 | Statement on COVID-19 | Statement | Research |
| 66 | US National Academics of Sciences and Medicine. | 2020 | The critical need for international cooperation during the COVID-19 pandemic: joint statement of Academies of Sciences and Medicine. | Statement | Epidemiology, Viral genomic, Research |
| 67 | van Roode M, dos Santos Ribeiro C, Farag E, Ahmed M, Moustafa A, van de Burgwal L, Claassen E, Nour M, Haringhuizen G, Koopmans M. | 2018 | Data sharing in public health emergencies: analysis of barriers and enablers from an outbreak response perspective (SHARE) | Report | Epidemiology, Research, and Viral genomic |
| 68 | Vong S, O'Leary M, Feng ZJ. | 2014 | Early response to the emergence of influenza A(H7N9) virus in humans in China: the central role of prompt information sharing and public communication | Letter | Research |
| 69 | Watson, J. on behalf of 182 signatories | 2020 | An open letter to Mehra et al and The Lancet. | Statement | Research |
| 70 | Wellcome/DfID workshop | 2018 | Data sharing in public health emergencies: learning lessons  from past outbreaks | Report | Epidemiology, Clinical, Research, and Viral genomic |
| 71 | Whitty CJM, Mundel T, Farrar J, Heymann DL, Davies SC, Walport MJ. | 2015 | Providing incentives to share data early in health  emergencies: the role of journal editors | Commentary | Research |
| 72 | WHO | 2011 | Pandemic influenza preparedness (PIP) Framework | Statement | Viral Genomic |
| 73 | Wilkinson MD, Dumontier M, Aalbersberg IJ, Appleton G, Axton M, Baak A, Blomberg N, Boiten J-W, da Silva Santos LB, Bourne PE, Bouwman, J, Brookes AJ, Clark T, Crosas M, Dillo I, Dumon O, Edmunds S, Evelo CT, Finkers R, Gonzalez-Beltran A, Gray AJG, Groth P, Goble C, Grethe JS, Heringa J, Hoen PACT Hooft R, Kuhn T, Kok R, Kok J, Lushner SJ, Martone ME, Mons A, Packer AL, Persson B, Sengstag T Slater T, Strawn G, Swertz MA, Thompson M, van der lei J, van Mulligan E, Velterop J, Waagmeester A, Wittenburg P, Wolstencroft K, Zhao J, Mons B. | 2016 | The FAIR Guiding  Principles for scientific data  management and stewardship | Article | Research |
| 74 | Wise, J. | 2020 | Data transparency- nothing has changed since tamiflu | News | Research |
| 75 | Worby CJ,  Lipsitch M, Hanage WP. | 2017 | Shared genomic variants: identification of transmission routes using pathogen deep-sequence data | Letter | Epidemiology |
| 76 | World Health Organization. | 2016 | Guidance for managing ethical issues in infectious disease outbreaks | Guidance and Policy | Epidemiology, Clinical, Research, and Viral genomic |
| 77 | World Health Organization. | 2007 | Ethical considerations in developing a public health response to pandemic influenza. | Guidance and Policy | Viral genomic |
| 78 | Xu B, Kraemer MUG, Open C-DCG. | 2020 | Open access epidemiological data from the COVID-19 outbreak | Letter | Research |
| 79 | Yozwiak NL, Schaffner SF Sabeti PC. | 2015 | Data sharing- make outbreak research open access | Commentary | Research |
